# Levels of soluble complement regulators predict severity of COVID-19 symptoms

**DOI:** 10.3389/fimmu.2022.1032331

**Published:** 2022-10-18

**Authors:** Anna L. Tierney, Wajd Mohammed Alali, Thomas Scott, Karen S. Rees-Unwin, CITIID-NIHR BioResource COVID-19 Collaboration, Stephen Baker, Simon J. Clark, Richard D. Unwin

**Affiliations:** ^1^ Division of Cardiovascular Sciences, School of Medicine, Faculty of Biology Medicine and Health, The University of Manchester, Manchester, United Kingdom; ^2^ Stoller Biomarker Discovery Centre and Division of Cancer Sciences, School of Medicine, Faculty of Biology Medicine and Health, The University of Manchester, Manchester, United Kingdom; ^3^ NIHR BioResource, Cambridge University Hospitals NHS Foundation Trust, Cambridge Biomedical Campus, Cambridge, United Kingdom; ^4^ Institute for Opthalmic Research is based at Eberhard Karls University of Tubingen, Tubingen, BW, Germany; ^5^ University Eye Clinic, Eberhard Karls University of Tubingen, Tubingen, BW, Germany; ^6^ Lydia Becker Institute of Immunology and Inflammation, Faculty of Biology, Medicine, and Health, University of Manchester, Manchester, United Kingdom

**Keywords:** COVID-19, SARS-CoV-2, complement, factor H, factor H-related proteins, biomarkers, mass spectrometry

## Abstract

The SARS-CoV-2 virus continues to cause significant morbidity and mortality worldwide from COVID-19. One of the major challenges of patient management is the broad range of symptoms observed. While the majority of individuals experience relatively mild disease, a significant minority of patients require hospitalisation, with COVID-19 still proving fatal for some. As such, there remains a desperate need to better understand what drives this severe disease, both in terms of the underlying biology, but also to potentially predict at diagnosis which patients are likely to require further interventions, thus enabling better outcomes for both patients and healthcare systems. Several lines of evidence have pointed to dysregulation of the complement cascade as a major factor in severe COVID-19 outcomes. How this is underpinned mechanistically is not known. Here, we have focussed on the role of the soluble complement regulators Complement Factor H (FH), its splice variant Factor H-like 1 (FHL-1) and five Factor H-Related proteins (FHR1-5). Using a targeted mass spectrometry approach, we quantified these proteins in a cohort of 188 plasma samples from controls and SARS-CoV-2 patients taken at diagnosis. This analysis revealed significant elevations in all FHR proteins, but not FH, in patients with more severe disease, particularly FHR2 and FHR5 (FHR2: 1.97-fold, p<0.0001; FHR5: 2.4-fold, p<0.0001). Furthermore, for a subset of 77 SARS-CoV-2 +ve patients we also analysed time course samples taken approximately 28 days post-diagnosis. Here, we see complement regulator levels drop in all individuals with asymptomatic or mild disease, but regulators remain high in those with more severe outcomes, with elevations in FHR2 over baseline levels in this group. These data support the hypothesis that elevation of circulating levels of the FHR family of proteins could predict disease severity in COVID-19 patients, and that the duration of elevation (or lack of immune activation resolution) may be partly responsible for driving poor outcomes in COVID-19.

## Introduction

The outbreak of the severe acute respiratory syndrome coronavirus 2 (SARS-CoV-2), which causes coronavirus disease 2019 (COVID-19), has caused an unprecedented global health emergency. As of August 2022, there have been more than 590 million cases of COVID-19 worldwide and more than 6.4 million deaths have been confirmed (https://covid19.who.int/). The clinical manifestation of COVID-19 infection presents with variable severity, ranging from asymptomatic infection, severe viral pneumonia, and even death.

SARS-CoV-2 is transmitted *via* respiratory droplets into the respiratory tract, where it infects host cells through the binding of angiotensin-converting enzyme 2 (ACE-2). ACE-2 is highly expressed on alveolar epithelial cells in the lungs, and other organs in the cardiovascular and renal systems. It is believed that direct damage to the former from the cytopathic replication of SARS-CoV-2 is a prime driver of COVID-19 pathology (1). Most individuals (~80%) experience mild symptoms that commonly include a fever, cough, fatigue, dyspnoea, myalgia, intestinal symptoms, and anosmia, which resolve after a short period (1). In a minority of patients (~15%), dyspnoea deteriorates, requiring hospital treatment. Within approximately six days this can intensify into viral pneumonia and acute respiratory distress syndrome (ARDS). Intensive efforts by the medical and scientific research community have resulted in the identification of life-saving treatments like dexamethasone and the rapid generation of several highly effective COVID-19 vaccines (2, 3). However, patients continue to be hospitalised with severe COVID-19 – especially in populations with low vaccine penetrance (4). As such, there remains a pressing need to identify prognostic biomarkers for COVID-19, to inform treatment decisions and allow more effective use of overstretched clinical resources.

A promising method for the discovery of disease prognostic biomarkers is liquid chromatography-mass spectrometry (LC-MS). This enables the relative levels of hundreds of plasma or serum proteins to be compared within clinically-derived samples, and provides candidate biomarkers whose levels differ between experimental groups. These differences may subsequently have predictive power in respect of diagnosis, disease stratification and prognosis. To date, a number of proteomics screening studies have been performed in various COVID-19 cohorts (5–13). While these differ in respect of cohort, study aim and method, a common finding between these is dysregulation of proteins in the complement cascade, albeit represented by different individual proteins in different studies.

This finding fits with other data where complement activation is known to contribute to ARDS in other viral diseases (14). In COVID-19, gene variants in the complement pathway, specifically *CD59*, *CFH* and *C4BPa* are associated with poorer outcomes (15) and targeted studies have demonstrated elevations of C5a and C5b-9 in plasma from individuals with moderate and severe disease (16). Together, these observations have led to suggestions that the complement cascade is a strong candidate target for therapies which may protect against severe outcomes (17).

The complement cascade can be activated *via* three pathways, the lectin, classical or alternative pathways, and it is likely that SARS-CoV-2 can activate several of these. The viral spike protein can activate complement *via* MASP1 binding (18), while several studies have shown increased classical pathway receptors e.g. C1q in severe disease (19, 20). However, all of these pathways converge onto a central amplification loop, where C3 is activated *via* cleavage to C3a and C3b. C3a acts as a powerful anaphylatoxin, while C3b is both an opsonin and forms complexes which subsequently result in the cleavage of C5 to produce C5a (a second anaphylatoxin) and C5b, initiating the formation of the C5b-9 membrane attack complex. The activation loop is regulated by inactivation of C3b by Factor I (FI) and one of its necessary co-factors (such as Factor H (FH)). The effects of COVID-19 on different aspects of this pathway are reviewed in (17).

In recent years, a series of five additional FH-related regulators have been identified (FHR1-5) and, along with a shorter splice isoform of FH called Factor H-Like 1 (FHL-1), have been shown to further modulate complement cascade activity. While FHL-1 retains the same function as its larger FH counterpart, the FHR proteins are FH/FHL-1 antagonists and drive complement activation instead of inhibition (21). Altered levels of these proteins play a key role in several complement-related diseases, including renal diseases such as atypical haemolytic uremic syndrome (aHUS), C3 glomerulopathy (C3G) (22, 23) and Age-Related Macular Degeneration (AMD) (24). FHRs 2 and 5 have also been identified as potentially differentially expressed in association with severe COVID-19 in global proteomics studies (12, 25).

Here, we hypothesised that complement dysregulation associated with COVID-19 could be a result of altered regulation of the amplification loop by FHR levels. Because FHRs are closely related in terms of sequence – indeed FHL-1 is identical to the first third of FH with the exception of a unique 4 amino acid C-terminal tail – they have not been measured together previously in COVID-19 cohorts. In this study, we measured the levels of all seven fluid-phase cofactors (FH, FHL-1 and FHR1-5) using a bespoke targeted mass spectrometry approach which can confidently and specifically identify and quantify these proteins in COVID-19 cohorts, both in baseline samples and a subset of longitudinal samples.

## Materials and methods

### Plasma samples and patient details

Ethical approval for this study was granted by the Cambridge Central Research Ethics Committee (17/EE/0025). All plasma samples were procured from the National Institute for Health and Care Research (NIHR) Bioresource. Participants attended multiple hospitals across the South-East of England with COVID-19 symptoms during the first wave of the pandemic in 2020 and recruited prospectively for inclusion within this study. A positive viral status was confirmed for all participants using PCR testing for SARS-CoV-2 RNA either in isolation or in combination with positive serology tests for anti-SARS-CoV-2 immunoglobulins. Participant status was stratified according to their clinical presentation, as shown in **Table 1**, with the approximate corresponding World Health Organisation (WHO) minimal common outcome scale given for comparison (26). Blood was collected in EDTA tubes (4ml). Samples were processed at a central BioResource service laboratory on the same day as receipt. EDTA blood tubes were centrifuged at 2500 rcf for 10 minutes with no brake. Plasma was then manually removed, and plasma stock aliquots were stored at -80°C until sub-aliquots were prepared for export for the research study.

**Table 1:** Patient cohort details and classification.

Group Nomenclature	Clinical Presentation	WHO Minimal Common Outcome Scale	# Individuals (Baseline)	# Individuals (Timecourse)
Control	Healthy Individuals	–	30	
NA Non-COVID	Symptomatic COVID-ve by PCR	–	46	
A ‘Asymptomatic’	Screening: no current symptoms	1	11	11
B ‘Mildly Symptomatic’	Screening: symptomatic, but not hospitalised	2-3	15	23
C ‘Moderate’	Hospitalised: oxygen not required	4	25	14
D ‘Severe’	Hospitalised: low-flow oxygen required	5-6	28	17
E ‘Critical	Hospitalised: Assisted ventilation required	7-9	33	12
**Total**			188	77

### Preparation of FH, FHL-1 and FHR1-5 standard peptides

Proteins of interest were measured using a targeted mass spectrometry approach as described in Cipriani et al. (24). Briefly, heavy isotope labelled peptide standards were obtained from Cambridge Research Biochemicals, Cambridge, UK. The peptide sequences for FH, FHL-1, FHR-1, FHR-2b, FHR-3, FHR-4 and FHR-5 were VTY**K**cFE, NGWSPTP**R**cIRVSFTL, ATFcD**F**PKINHGILYDEE, AM**F**cDFPKINHGILYDEE, VAcHPG**Y**GLP**K**AQTTVTcTE, **Y**QcQSYYE, and **R**GWSTPPIcSFT**K**GE, respectively. A bold type denotes those residues that contained isotopically heavy amino acid, where K (+8), R (+10), F (+10) and Y (+10). A standard peptide stock was made in 105 μL in 0.1% TFA. The final concentration was 47.6 ng/µL for FH; 0.95 ng/µL for FHL-1; 7.14 ng/µL for FHR-1; 19 ng/µL for FHR-2b; and 4.76 ng/µL for FHR-3, FHR-4 and FHR-5. This concentrated standard mixture was subsequently aliquoted into 5 µL and stored at -80°C.

### Plasma sample preparation for LC-MS/MS analysis

Frozen plasma samples were thawed to room temperature, vortexed for 5 min and centrifuged for 30 min at 13,300 g and 5 μL aliquoted to be processed. To each sample 90 μL, 1 μL, and 2 μL of 50 mM ammonium bicarbonate (pH 7.8), 500 mM dithiothreitol (prepared in 50 mM ammonium bicarbonate), and 1% w/v ProteaseMAX™ solution (Promega, Southampton, UK), prepared in 50 mM ammonium bicarbonate, were added, respectively and incubated for 25 min at 56°C. The samples were allowed to cool at room temperature, followed by the addition of 3 μL 500 mM iodoacetamide in 50 mM ammonium bicarbonate and incubated in the dark at room temperature for 15 mins. Proteins were then digested by addition of 43 μL and 1 μL of 50 mM ammonium bicarbonate (pH 7.8), and 1% w/v ProteaseMAX™ solution in 50 mM ammonium bicarbonate, respectively, with 2.5 μL endoproteinase Glu-C (1μg/μL), followed by incubation at 25°C for 16 h with shaking at 400 rpm.

Peptide standards were diluted by adding 195 μL of 50:50 acetonitrile:water to the 5 μL of concentrated standard mixture, and 2 μL of this diluted standard was added to each sample alongside 6 μL of 10% v/v TFA. By this point, the standard peptides are at the final concentration of 500 nM, 5 nM, 32.75 nM, 86.75 nM, 21.68 nM, 41.5 nM and 27.5 nM, for FH, FHL-1 and FHR-1–FHR-5, respectively. The samples were dried in a centrifugal evaporator (Eppendorf), and reconstituted in 50 μL of 0.1% v/v TFA and transferred to LC autosampler vials for analysis by LC-MS/MS.

### Liquid chromatography-mass spectrometry analysis

The analysis was conducted in two parts. First, baseline samples were randomized into batches, with each batch containing 20 experimental samples with roughly equal numbers from each group and a set of quality control (QC) samples. These QC samples were duplicates of a commercial, standard human serum sample (Sigma), a duplicate of one of the samples in the batch, and a replicate from a previously run duplicate sample. Subsequently, paired samples where a 28d sample was available were analysed, including a repeat of the baseline sample along with its 28d paired sample. Randomisation and batch structure were the same across both studies.

All samples were analysed by Liquid Chromatography-Selected Reaction Monitoring-Mass Spectrometry (LC-SRM-MS) using the method described in (24). Using a 6495 triple-quadrupole mass spectrometer with an electrospray ion source (Agilent), connected to an Agilent Infinity 1200 Series liquid chromatography system, 4 μL of each sample was injected directly onto a C18 column (250 mm × 2.1 mm I.D., Thermo Scientific Acclaim 120, 3 μm particle size) held at 50°C. Peptides were eluted at a 250 μL/min flow rate using the previously described gradient (24). Six acquisition windows were used for the eluting peptides, where one window was acquired for both FHL-1 and FHR-2.

### Data analysis

Selected Reaction Monitoring (SRM) data were processed using Skyline (v19.1.0.193) (27). The retention time and heavy peptide peak area were visually checked for all samples to verify that the peaks were allocated and integrated appropriately. Peak-area data was extracted from Skyline into Microsoft Excel where the peak areas of heavy and light transitions were compared. The largest signal was used to quantify on-column loading of the endogenous peptide, whereas the other two transitions were used as a qualification signal to ensure the specificity and agreement in quantitation. Finally, concentration per unit volume was calculated based on each sample injection containing the equivalent of 0.8 μL plasma. All further statistical analysis was performed in GraphPad Prism (version 9.2.0). Initial normality testing was carried out on all datasets using a Kolmogorov-Smirnov test, with the most appropriate test then being used for regression analyses as described in the text.

## Results

### Measurement of FH, FHL-1 and FHR1-5 in baseline samples

Baseline samples were collected from 188 individuals and categorised according to COVID-19 diagnosis and severity. Key among these patients are those who were non-symptomatic and SARS-Cov-2 negative (control, n=30), and those who attended for SARS-Cov-2 testing and were therefore displaying symptoms, but were tested as SARS-Cov-2 negative (NA, n=46). Demographic data for these individuals is shown in **Table 2**.

**Table 2:** Demographic data for patients enrolled in baseline measurement study.

Patient Classification	Total	Age (yrs) Mean (range) s.d (n)^a^	BMIMean (range) s.d (n)^a^	% Male^a^
Ctrl	30	42.0 (20-73) 16.8 (n=30)	26.3 (23-30.1) 3.0 (n=4)	60% (12/20)
NA	46	57.4 (18-89) 18.13 (n=46)	28.0 (18.3-53.9) 8.1 (n=42)	43.5% (20/46)
A	11	39.4 (20-72) 15.6 (n=11)	25.4 (20.9-31.8) 3.8 (n=8)	18.2% (2/11)
B	15	36.47 (23-58) 12.0 (n=15)	27.0 (19.6-43.4) 6.8 (n=13)	13.3% (2/15)
C	25	59.4 (18-87) 16.5 (n=24)	29.3 (20-47.9) 7.2 (n=22)	41.6% (10/24)
D	28	66.2 (39-87) 14.3 (n=26)	29.0 (15-42.4) 5.6 (n=24)	69.2% (18/26)
E	33	62.8 (26-86) 13.82 (n=33)	32.0 (22.5-40.2) 5.3 (n=22)	67.9% (19/28)

a Due to the nature of the cohort, not all demographic information is available for all participants. The number of values on which the reported summary data for each characteristic is based is therefore provided.

Samples were randomised into 11 analytical batches, with each batch containing 2 types of quality control samples, one being a duplicate analysis of a single quality control sample in every batch, the second being a duplicate of an experimental sample which was analysed a third time in the subsequent batch. Analysis of the common quality control sample showed a batch effect for measurement of FH in batches 2, 3 and 5, for FHR-2 in the 3rd batch and for FHR4 in batches 1 and 2. Data for these proteins from these batches were subsequently excluded from further analysis. The variability of the QC in remaining batches was plotted on Levey-Jennings plots (**Supplementary Figure 1**) and percent coefficient of variance (%CV) measured across all batches was FH = 17.0%, FHL-1 = 14.3%, FHR1 = 14.7%, FHR2 = 17.8%, FHR3 = 10.3%, FHR4 = 23.5% and FHR5 = 13.0%. Evaluation of triplicate COVID-19 samples across adjacent batches, demonstrated that 93.4% of the measurements have a %CV less than 25% (**Supplementary Table 1**), indicating high assay reproducibility.

**Figure 1:**
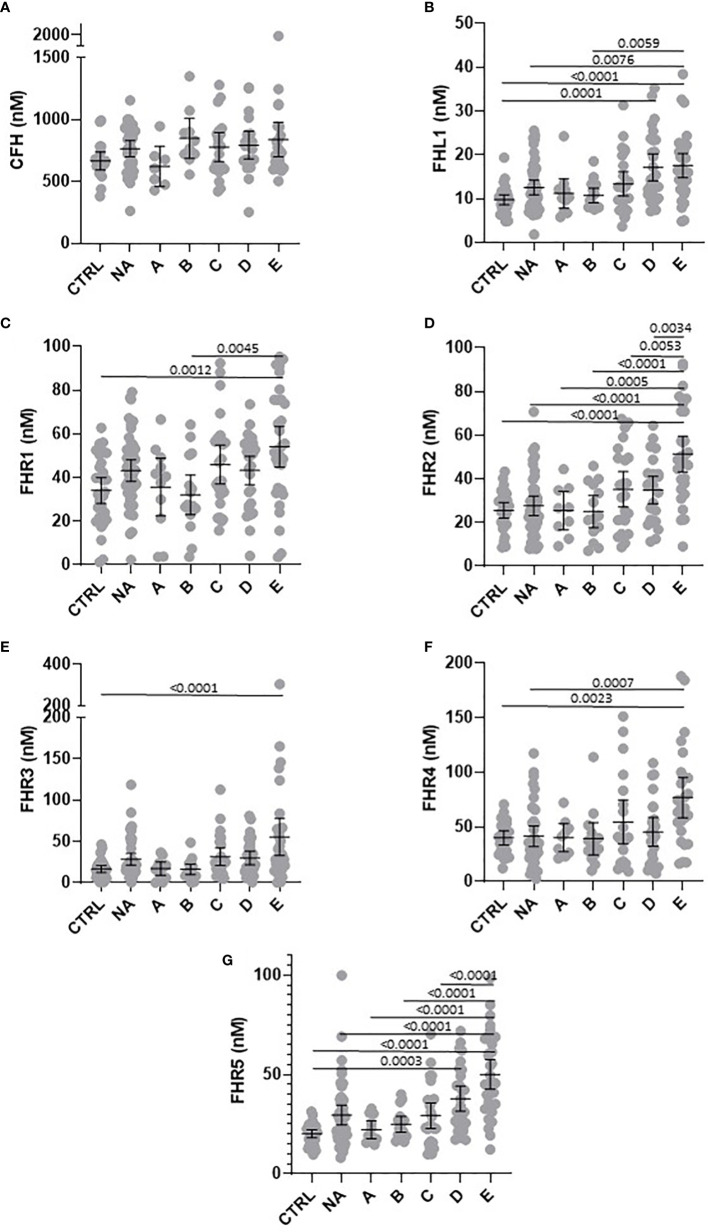
Circulating concentration of FHL-1 and FHR1 to FHR5 is elevated in COVID-19 patients. Scatter plots of FH **(A)**, FHL1 **(B)**, and FHR1 to FHR5 **(C–G)** represent the measured plasma protein concentration in nM. Pairwise adjusted p-values were obtained from a Tukey’s multiple comparisons test (4 d.p.).

To compare levels of circulating FH, FHL-1 and FHR between groups, we confirmed normality of distribution in most groups using a Kolmogorov-Smirnov test and data were analysed *via* a one-way ANOVA. Differences in circulating proteins associated with COVID-19 severity were identified for FHL-1 (p<0.0001), FHR1 (p=0.0007), FHR2 (p<0.0001), FHR3 (p<0.0001), FHR4 (p=0.0006) and FHR5 (p<0.0001). FH concentrations did not correlate with severity (p=0.126). Pairwise comparisons between groups were subsequently carried out using a Tukey’s multiple comparison test. All associations with adj. p<0.01 are described in **Figure 1**. Notably there were significant elevations in circulating levels of all proteins comparing controls and severe disease groups. These differences were greatest for FHR2 and FHR5 in particular, both of which saw a greater than 2-fold increase in protein levels across these groups, and indeed significant differences between almost all other groups and the most severe patients. These data suggest that FHL-1 and FHR levels are related to disease severity.

**Figure 2:**
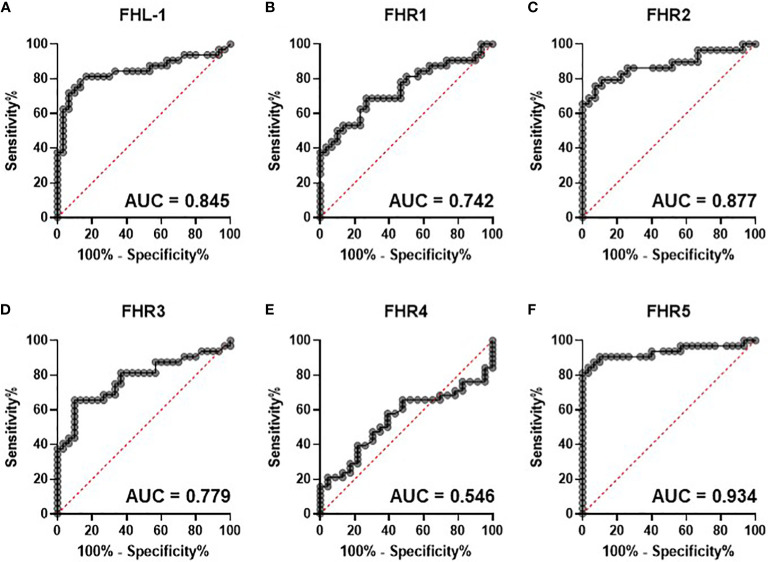
Circulating complement cofactors predict disease severity. Receiver Operating Characteristic (ROC) curves for control versus severe [group **(E)**] disease are shown for circulating FHL-1 **(A)** and FHRs 1-5 **(B-F)**. Area under the curve is provided to 3 d.p.

To test this further we calculated individual ROC curves for all proteins to demonstrate the predictive power of the FHR proteins with respect of severe disease (**Figure 2**). As expected FHR2 and FHR5 demonstrated the greatest power to predict the most severe disease vs controls, with AUROC values of 0.877 and 0.943 respectively.

In this baseline cohort, we noted that age was significantly different, with groups A and B being generally younger than other groups. This is demonstrated in **Supplementary Figure 2A**, where age distributions can be seen (although age ranges are similar). To rule this out as a factor driving the observed differences, we studied the relationships between age and FH, FHL-1 and FHR1-5 levels in the control and NA cohorts (n=76, where age data were available). No significant associations were seen (**Supplementary Figure 2B-H**), confirming that the observed elevation in protein levels with more severe COVID-19 symptoms is disease, rather than age-driven. The cohort also shows some variability in sex in the experimental groups, although the critical comparisons of Control versus groups C, D and E are well aligned. Nevertheless, we also investigated the possibility of sex-associated differences for all protein levels in the control and NA cohorts. No such statistically significant association was found (**Supplementary Figure 3**).

### Measurement of FH, FHL-1 and FHR1-5 in 28 day samples

For 77 SARS-CoV-2 positive individuals, we collected an additional sample at approximately 28 days after the first. Of these 77 samples, 58 were included in the baseline analysis, with a further 19 analysed only in this timecourse study. The demographic data for this subset is shown in **Table 3**. Notably, while most samples were collected between 25-31 days after the first sample, there are 2 cases in Group C (49 and 53 days), 4 in Group D (40, 44, 47 and 48 days) and one in Group E (46 days) where samples were collected over 40 days later. This does not cause a significant difference in sampling time between groups.

**Table 3:** Demographic data from patient subset used in time course analysis.

Patient Classification	Total	Age (yrs) Mean (range) s.d (n)^a^	BMI Mean (range) s.d (n)^a^	% Male^a^
A	11	37.7 (20 - 72) 15.9 (n = 11)	25.2 (20.9-31.8) 3.9 (n = 8)	18.2% (2/11)
B	23	39.0 (20 - 70) 13.9 (n = 23)	25.9 (19.6 - 43.4) 6.1 (n = 19)	13% (3/23)
C	14	59.8 (42 - 77) 11.0 (n = 13)	30.3 (21.1 - 42.9) 6.7 (n = 12)	53.9% (7/13)
D	17	63.1 (44 - 87) 12.0 (n = 17)	29.2 (20.8 - 36.2) 4.5 (n = 16)	64.7% (11/17)
E	12	62.3 (38 - 81) 12.4 (n = 12)	32.3 (22.5-40.2) 6.1 (n = 10)	75% (9/12)

a Due to the nature of the cohort, not all demographic information is available for all participants. The number of values on which the reported summary data for each characteristic is based is therefore provided.

Later timepoint samples were batched alongside the baseline samples for each individual and randomly assigned to analysis batches for measurement of circulating FH, FHL-1 and FHR1-5 proteins. Similar cross-batch quality controls were employed as with the baseline study. Reproducibility across batches was excellent, with replicates of a standard samples run in all batches routinely measuring within 2 s.d. of the mean (**Supplementary Figure 4**), with replicates of an individual study sample analysed across consecutive batches FH = 11.2%, FHL-1 = 16.2%, FHR1 = 28.6%, FHR2 = 9.1%, FHR3 = 9.45%, FHR4 = 3.8% and FHR5 = 6.3% (1 d.p.).

This study contains a nested, independent replicate analysis of the baseline samples, albeit with an overlapping subset of samples. However, analysis of this subset confirms the finding from the first, larger study, in that statistically significant elevations of FHL-1 (p=0.047), FHR3 (p=0.045) and FHR5 (p=0.0071) proteins could be observed, in this case between Group A and Group E individuals, while FHR2 showed a significant increase between Groups B and C (p=0.048) and Groups B and D (p=0.041), implying that elevations in FHL-1 and FHRs are apparent upon replicate analysis, albeit this is a lower powered subset. Note that this analysis did not include healthy controls or SARS-CoV-2 negative patients where the largest differences were seen in the original baseline study.

**Figure 3:**
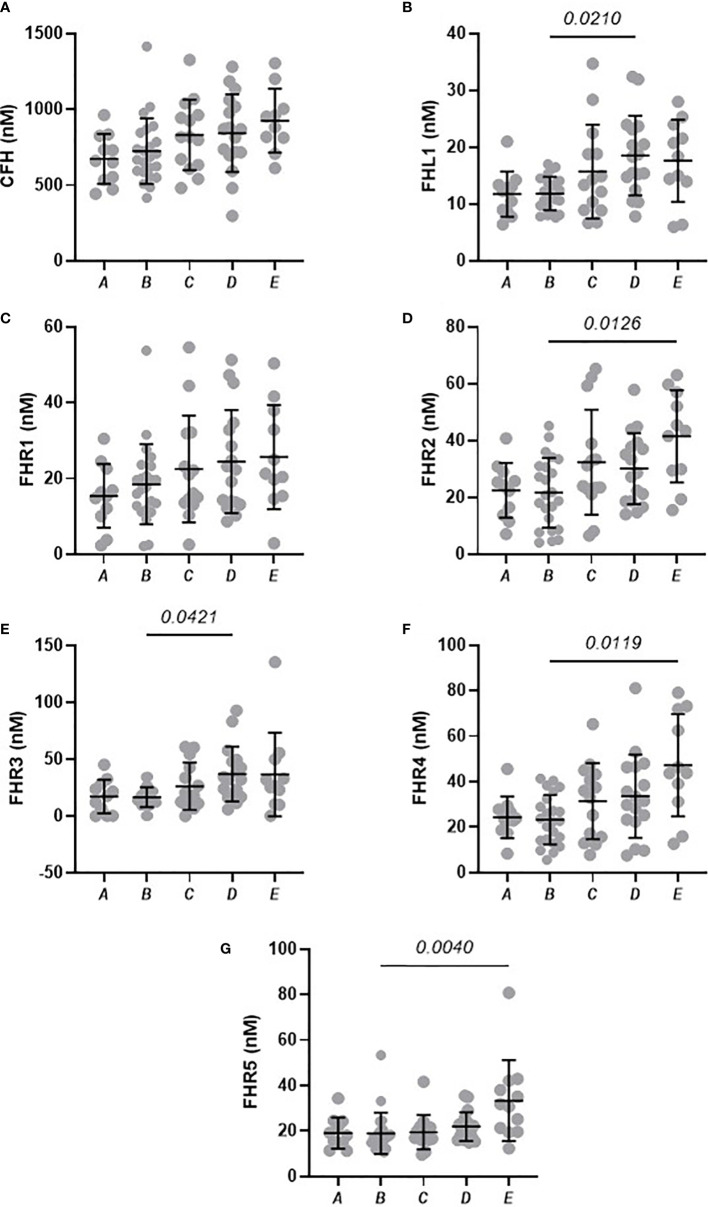
Circulating concentrations of FHL-1 and FHR1 to FHR5 in COVID-19 patients after 28 days. Scatter plots of FH **(A)**, FHL1 **(B)**, and FHR1 to FHR5 **(C–G)** represent the measured plasma protein concentration in nM. Pairwise adjusted p-values were obtained from a Tukey’s multiple comparisons test (4 d.p.).

Analysis of day 28 samples demonstrated that, although COVID-19 symptoms had largely resolved in most cases, with only 5 remaining in intensive care at this timepoint, there is still evidence of elevations of FHL-1 and FHR proteins at this time in the most severe cases (**Figure 3**), most notably FHL-1, FHR2 and FHR5.

**Figure 4:**
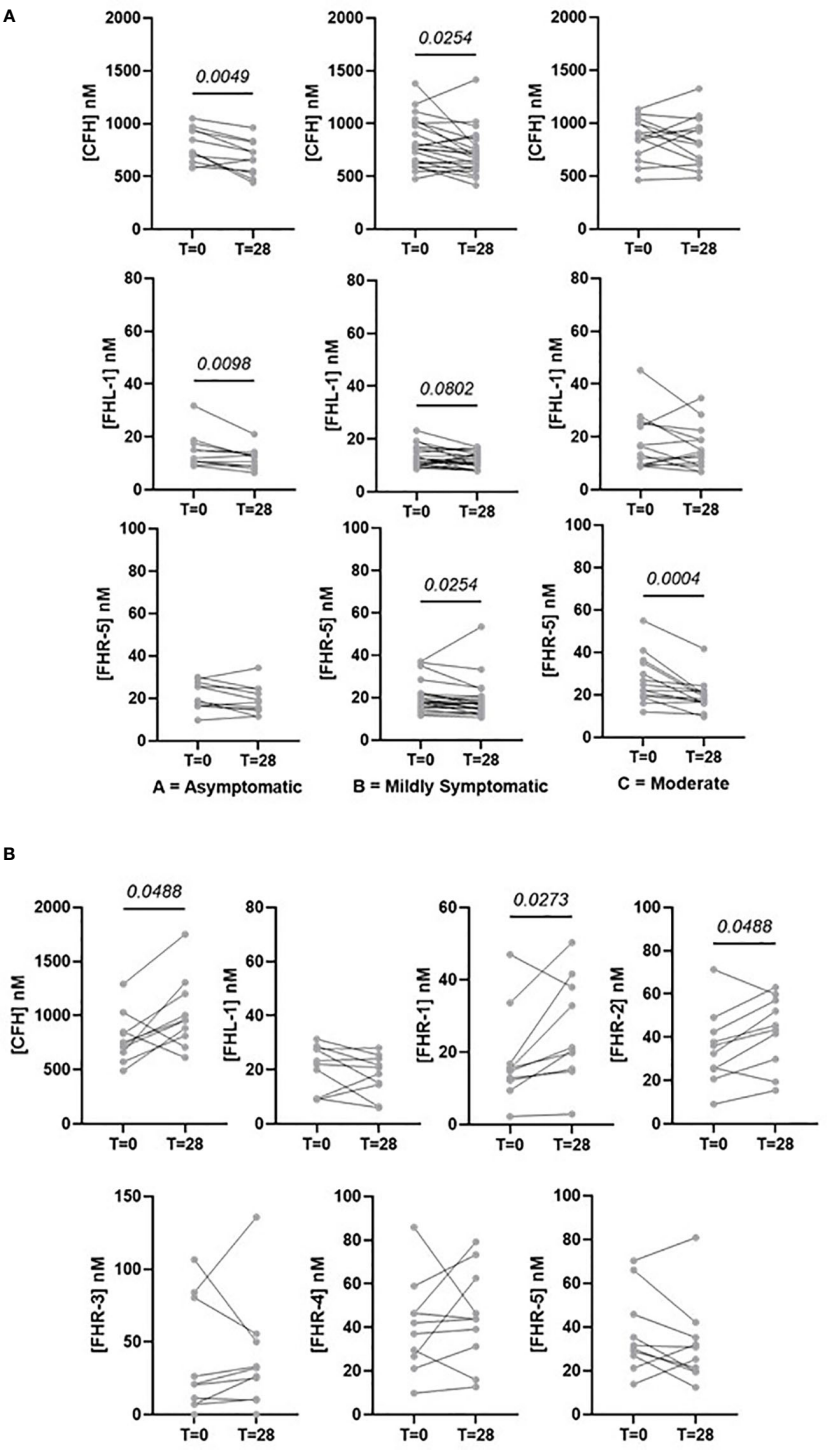
Selected pairwise comparisons of circulating concentrations of FHL-1 and FHR1 to FHR5 in COVID-19 patients after 28 days. **(A)** CFH, FHL-1 and FHR5 in asymptomatic to moderate symptomatic individuals. **(B)** CFH, FHL-1 and FHR1-5 in patients with critical (Group E) COVID-19.

Pairwise analysis of samples taken from the same individual across the two timepoints reveals how the levels of FH, FHL-1 and FHRs resolve over time. All comparisons for each protein separated by disease severity are shown in **Supplementary Figure 4**. As may be expected, there is a general decrease in levels of all proteins after 28 days in those individuals with asymptomatic, mild or moderate symptoms as in the majority of cases symptoms resolve. This is particularly evident for FHL-1 and FHR5. Notably, this is also the case for FH, implying that individuals do experience a slight elevation of FH level upon infection, although this is not detectable in a standard case-control design due to the background of between-patient variability (**Figure 4A**). In contrast, individuals who experienced severe COVID-19 symptoms demonstrate a further elevation in circulating FH, FHR1 and FHR2 levels 28 days after their initial diagnosis, on top of the higher levels of these proteins seen in these patients versus a healthy cohort in our baseline analysis. (**Figure 4B**). Strikingly, 9/12 (75%) of Group E samples showed a greater than 50% elevation in at least one of the FHRs after 28 days, compared to 14/65 (22.5%) in all other groups combined. However, this continued elevation was not correlated with length of stay in intensive care, or whether the patient was still in intensive care at this second timepoint, although we do not have data regarding other symptom severity at this time, e.g. non-ICU admission or continued requirement for oxygen at home. These data support the hypothesis that levels of circulating soluble complement regulators play a role in determining and/or driving disease severity in patients with COVID-19.

## Discussion

Immune overactivation is established as a key driver of COVID-19 disease severity, although the mechanisms underpinning this overactivation are unclear. While much of the focus has been on the adaptive response, there is clearly a role for the activation of the complement system in driving, or contributing to this overactivation (17). In this study, we sought to uncover the role played by a key series of complement activation regulators, the Complement Factor H family. FH is a critical regulator of the complement amplification loop, as a co-factor for Factor I it is required for the degradation and inactivation of C3b. A splice variant of FH, FHL-1, has the same activity but is present at much lower circulating levels, while the FHR proteins, encoded by distinct genes at the same chromosomal location, retain C3b binding capability but lack co-factor activity, and so can compete with FH and in essence prevent C3b breakdown. We hypothesised that, given existing data on complement overactivation and the observation from global proteomics studies that members of the complement family are overactivated in severe disease, levels of this family of co-factors could play a role in predicting disease severity or outcome in SARS-Cov-2 positive patients.

To measure these proteins, we used a mass spectrometry-based assay. Because MS can accurately identify peptides based on their mass, it provides enhanced selectivity over antibody-based approaches, which is especially important in studies such as this where the target proteins share significant homology. While antibody assays have been published for some FHRs (28), no such assay has been developed to detect FHL-1, which is identical to the N-terminal part of FH with the exception of a 4 amino acid C-terminal tail (29). This approach has been used previously to study complement co-factors in age-related macular degeneration (24) and glioblastoma (30). A standard quality control regimen was used to ensure that all samples were randomised across analysis batches, and common samples included to ensure that data were comparable between batches

Our study was divided into two independent parts. The first was an analysis of samples collected at baseline (time of diagnosis) and included both a healthy control cohort and a cohort who were symptomatic and presented at hospital for a SARS-CoV-2 test but were found to be negative. The second was a time course analysis of SARS-CoV-2-positive individual approximately 28 days following initial diagnosis.

The baseline study identified that all measured proteins, with the exception of FH, were present at higher levels in more severe disease. Elevation of FHL-1, independent of FH, is particularly striking since FHL-1 is a spice variant and as such is driven from the same promoter as FH, implying an altered regulation of splicing, translation or degradation of this shorter isoform compared to its larger and more abundant counterpart, an observation that we have also made in age-related macular degeneration (24) and glioblastoma (30). While increases were seen in all FHR proteins, elevations were highest in FHR2 and FHR5, which increased between healthy controls and severe disease by 1.97-fold (p<0.0001) and 2.4-fold (p<0.0001) respectively. ROC curves constructed between severe COVID-19 vs healthy controls gave AUROC values of 0.877 and 0.934 suggesting a strong predictive relationship. ROC curves considering multiple proteins did not improve this prediction significantly, while ROC curves on all COVID-19 patients to predict hospitalisation yielded more modest AUROCs of 0.76.

While previous studies of the global proteome have incidentally detected elevation in FHR-5 correlated with more severe cases of COVID-19 (12), this study represents the first targeted analysis of these proteins and as such is the first observation of a global increase across the factor H family of proteins. These data compare favourably to other recent studies investigating COVID-19 severity biomarkers. For example, the recent work by Wang et al. (31) used a 30 protein panel to predict disease severity and using a machine learning approach achieved AUROC for severe disease (WHO outcome score 7) vs mild (WHO outcome score 3) of approximately 0.85. In our study a similar analysis for FHR5 only, between groups A+B vs Group D+E, gives a AUROC of 0.84 for similar sized cohorts. Intriguingly, FHR2, but not FHR5, was identified in that group’s discovery proteomics effort (13) but was not included in the final panel of 30.

The second part of this study looked at how complement regulator levels change over time, with a subset of individuals providing a second sample at approximately 28 days post the baseline sample. Paired analysis of samples from individuals with symptomatic, mild or moderate disease (i.e. disease not requiring hospitalisation) demonstrated a reduction in levels of most FH-family proteins, including FH which we did not show to be elevated in our baseline study. This is possibly due to the magnitude of change being relatively small compared to between patient variability, so it is masked in a case-control design but is apparent in a time course where each individual acts as their own control. This supports our observation from the baseline study that SAR-CoV-2 infection results in elevation of circulating complement co-factors. However, in individuals with severe disease co-factor levels are actually increased on average 28 days following diagnosis. It is unclear whether this is a true reflection of severe disease i.e. that complement can become ‘more dysregulated’ over time in more severe cases, or simply a consequence of our study taking few timepoint samples. A higher resolution timecourse, perhaps weekly sampling and with a more reproducible sampling frequency than was achieved in this cohort, is required to confirm the trajectory of these proteins more accurately. We also recognise that there is a sampling bias in this study, as patients with the most severe disease who did not survive for 28 days after diagnosis are necessarily excluded. What these data do show is that the complement cascade response is not resolved at 28 days in severe cases.

This study does have limitations. Due to the nature of the pandemic at the time of collection, complete metadata is not available for all individuals in the cohort as it was not collected at the time and is now only obtainable by manual extraction from patient records. As such it is not possible for us to perform convincing multivariate analysis or to correlate our findings with co-morbidities and treatments, for example. However, in previous studies on larger cohorts we have shown no effect of age, sex or BMI on FH, FHL-1 or FHR1-5 levels (24) and so we do not anticipate that adding these variables would alter our conclusions. Samples were collected in wave 1 of the pandemic in the UK, driven primarily by the original wild-type variant of the SARS-CoV-2 virus. All samples were collected before the emergence of the delta variant which caused a more severe disease (32) and the current predominant omicron variant which is more transmissible but appears to result in a less severe phenotype that delta, albeit worse than the wild-type, even in unvaccinated individuals (33). As such we believe it would be valuable to reanalyse FHL-1 and FHR levels in samples from both the delta and omicron waves, and from better defined cohorts in terms of both numbers and clinical metadata to investigate whether levels of these proteins contribute to the more severe symptoms seen with these variants, along with correlation of FHR levels with other complement activation or inflammatory markers in these cohorts.

Increased FHR levels are predicted to decrease C3b turnover, and as such increase complement cascade activation. This agrees with observations in clinical samples around increase deposition of C5b-9 (16, 20) in more severe cases and supports the hypothesis that the complement cascade is a viable target for both prediction of COVID-19 prognosis in infected individuals, and supports therapeutic efforts for complement blockade as a means of treating or preventing severe COVID-19 outcomes.

## Data availability statement

The raw data supporting the conclusions of this article will be made available by the authors, without undue reservation.

## Ethics statement

The studies involving human participants were reviewed and approved by Cambridge Central Research Ethics Committee. The patients/participants provided their written informed consent to participate in this study.

## Author contributions

RU and SC contributed to conception and design of the study. AT, WA, TS and RU acquired the data and performed initial analysis. KR-U analysed the clinical data. All authors interpreted the data. WA, TS and RU wrote the first draft of the manuscript. All authors contributed to the article and approved the submitted version.

## Funding

Development of the FH, FHL-1 and FHR assay was funded by the Medical Research Council, UK (MR/P025838/1). The Stoller Biomarker Discovery Centre was established with an award from the MRC (MR/M008959/1). The work was funded by awards from NIHR to the NIHR BioResource (RG94028 & RG85445). SJC receives money and support from the Helmut Ecker Foundation.

## Acknowledgments

We thank NIHR BioResource volunteers for their participation, and gratefully acknowledge NIHR BioResource centres, NHS Trusts and staff for their contribution. We thank the National Institute for Health and Care Research, NHS Blood and Transplant, and Health Data Research UK as part of the Digital Innovation Hub Programme. The views expressed are those of the author(s) and not necessarily those of the NHS, the NIHR or the Department of Health and Social Care. This work was facilitated by the Manchester NIHR Biomedical Research Centre and the Greater Manchester Comprehensive Local Research Network.

## Conflict of interest

SC and RU are inventors named in patent applications that describe the use of complement inhibitors for therapeutic purposes and the use of circulating complement protein measurement for patient stratification, and are co-founders of and shareholders in Complement Therapeutics, a company which focuses on the development of complement targeted therapeutics, for chronic diseases focussed on Age-related Macular Degeneration.

The remaining authors declare that the research was conducted in the absence of any commercial or financial relationships that could be constructed as a potential conflict of interest.

## Publisher’s note

All claims expressed in this article are solely those of the authors and do not necessarily represent those of their affiliated organizations, or those of the publisher, the editors and the reviewers. Any product that may be evaluated in this article, or claim that may be made by its manufacturer, is not guaranteed or endorsed by the publisher.
