# Supplementary material for: Levels of soluble complement regulators predict severity of COVID-19 symptoms
Source: Front Immunol. 2022 Oct 18;13:1032331. doi: 10.3389/fimmu.2022.1032331 (PMC9624227; doi:10.3389/fimmu.2022.1032331)
Supplement: Supplementary file 1 [file DataSheet_1.docx]

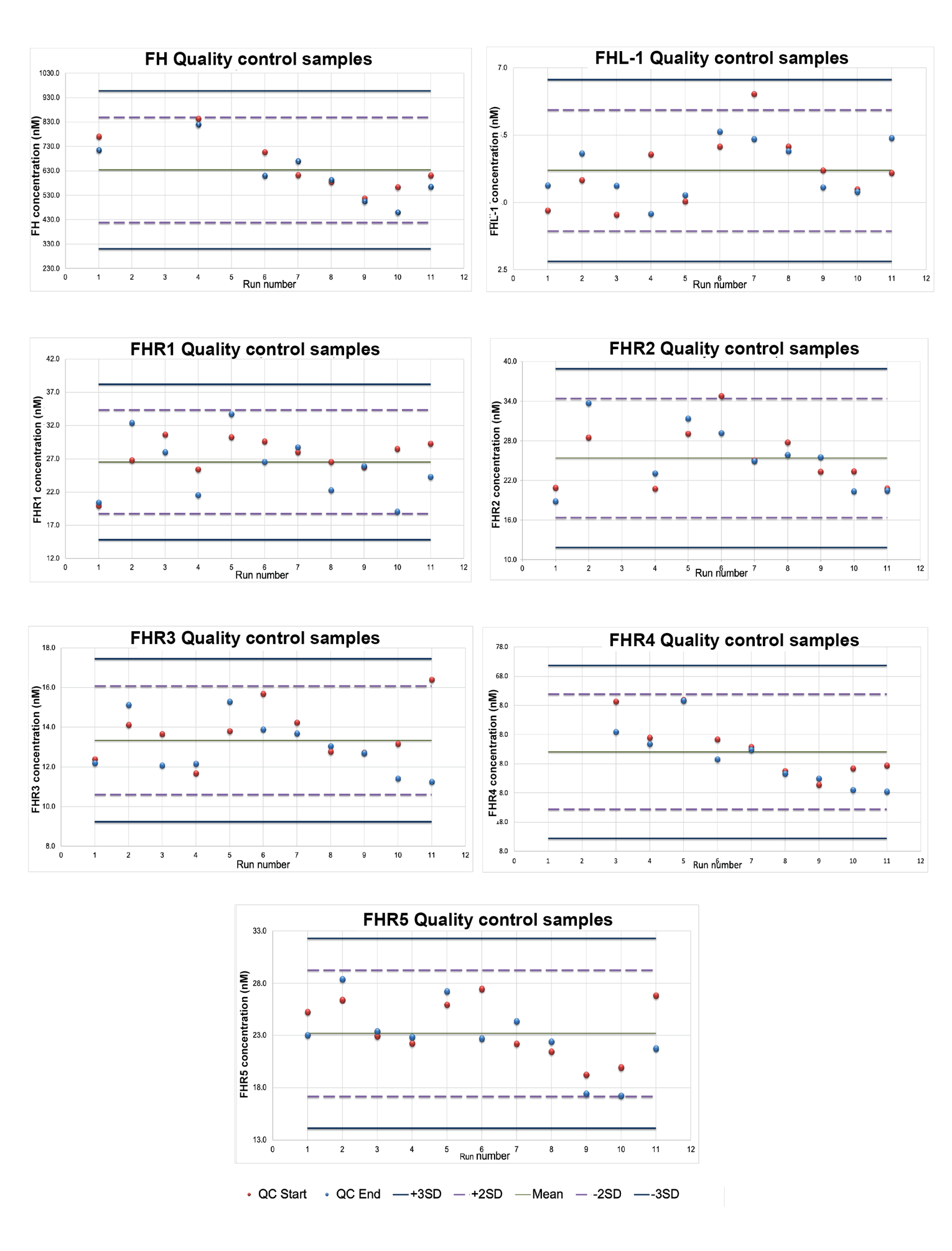


**Supplementary Figure 1.** **Levey-Jennings graphs illustrating the QC results to monitor the between-batch stability of the whole process across 12 batches for the analysis of 188 baseline samples.** Duplicate QC samples were included in each batch, and the concentration of each peptide was measured.

**Supplementary Table 1.** COVID sample cross batch replicate data. Selected samples were analysed twice in the same batch (Sample and Sample_Dup), and then again in the subsequent batch (Sample_Rep) to ensure batch-to-batch consistency.

| Batch |  | CFH | FHL-1 | FHR1 | FHR2 | FHR3 | FHR4 | FHR5 |
| --- | --- | --- | --- | --- | --- | --- | --- | --- |
| 1 | CV0929 | 540.7 | 4.9 | 29.2 | 19.4 | 19.3 |  | 11.7 |
| 1 | CV0929_Dup | 648.0 | 5.6 | 31.3 | 20.3 | 20.5 |  | 13.1 |
| 2 | CV0929_Rep | | 7.9 | 66.4 | 43.6 | 36.5 |  | 19.0 |
|  | Mean | 594.3 | 6.1 | 42.3 | 27.8 | 25.4 |  | 14.6 |
|  | SD | 75.9 | 1.6 | 20.9 | 13.7 | 9.6 |  | 3.9 |
|  | CV | 12.8 | 26.1 | 49.4 | 49.5 | 37.8 |  | 26.6 |
|  |  |  |  |  |  |  |  |  |
| 2 | CV0152 |  | 33.4 | 34.2 | 11.2 | 72.6 |  | 66.2 |
| 2 | CV0152_Dup | | 28.0 | 59.6 | 13.7 | 69.8 |  | 61.7 |
| 3 | CV0152_Rep | | 31.2 | 52.4 |  | 86.6 | 57.2 | 60.8 |
|  | Mean |  | 30.9 | 48.7 | 12.5 | 76.3 | 57.2 | 62.9 |
|  | SD |  | 2.7 | 13.1 | 1.8 | 9.0 |  | 2.9 |
|  | CV |  | 8.8 | 26.9 | 14.2 | 11.8 |  | 4.6 |
|  |  |  |  |  |  |  |  |  |
| 3 | CV0304 |  | 12.1 | 52.7 |  | 23.3 | 54.2 | 30.8 |
| 3 | CV0304_Dup | | 12.4 | 53.8 |  | 24.4 | 54.2 | 31.4 |
| 4 | CV0304_Rep_5 | | 11.8 | 58.8 | 31.5 | 28.4 | 60.4 | 37.6 |
|  | Mean |  | 12.1 | 55.1 | 31.5 | 25.3 | 56.3 | 33.3 |
|  | SD |  | 0.3 | 3.2 |  | 2.7 | 3.6 | 3.8 |
|  | CV |  | 2.4 | 5.9 |  | 10.8 | 6.4 | 11.3 |
|  |  |  |  |  |  |  |  |  |
| 4 | CV0274_5 | 539.3 | 24.4 | 27.2 | 10.7 | 61.5 | 35.1 | 25.1 |
| 4 | CV0274_Dup_5 | 514.7 | 22.7 | 28.8 | 16.7 | 55.5 | 31.7 | 24.9 |
| 5 | CV0274_Rep | | 25.9 | 66.7 | 12.8 | 55.5 | 33.3 | 25.6 |
|  | Mean | 527.0 | 24.3 | 40.9 | 13.4 | 57.5 | 33.3 | 25.2 |
|  | SD | 17.3 | 1.6 | 22.4 | 3.1 | 3.4 | 1.7 | 0.3 |
|  | CV |  | 6.5 | 54.7 | 22.8 | 5.9 | 5.1 | 1.4 |
|  |  |  |  |  |  |  |  |  |
| 5 | CV0180 |  | 29.7 | 37.4 | 64.7 | 0.1 | 83.6 | 75.4 |
| 5 | CV0180_Dup | | 25.2 | 33.3 | 65.6 | 0.3 | 90.1 | 77.0 |
| 6 | CV0180_Rep | 1039.1 | 27.7 | 4.2 | 73.6 | 6.8 | 62.7 | 70.1 |
|  | Mean | 1039.1 | 27.5 | 25.0 | 68.0 | 2.4 | 78.8 | 74.1 |
|  | SD |  | 2.3 | 18.1 | 4.9 | 3.8 | 14.3 | 3.6 |
|  | CV |  | 8.3 | 72.4 | 7.2 | 160.5 | 18.2 | 4.9 |
|  |  |  |  |  |  |  |  |  |
| 6 | CV0323 | 898.2 | 12.3 | 27.6 | 8.2 | 10.8 | 37.6 | 23.1 |
| 6 | CV0323_Dup | 763.1 | 12.6 | 22.3 | 7.2 | 12.1 | 30.3 | 19.1 |
| 7 | CV0323_Rep_8 | 792.2 | 11.9 | 27.4 | 5.9 | 9.9 | 31.4 | 21.4 |
|  | Mean | 817.8 | 12.3 | 25.8 | 7.1 | 10.9 | 33.1 | 21.2 |
|  | SD | 71.1 | 0.4 | 3.0 | 1.2 | 1.1 | 3.9 | 2.0 |
|  | CV | 8.7 | 3.1 | 11.5 | 16.3 | 10.3 | 11.9 | 9.4 |
|  |  |  |  |  |  |  |  |  |
| 7 | CV0045_8 | 878.8 | 10.1 | 32.1 | 31.4 | 25.1 | 40.4 | 22.8 |
| 7 | CV0045_Dup_8 | 849.6 | 9.3 | 27.0 | 33.8 | 22.0 | 42.8 | 22.5 |
| 8 | CV0045_Rep | 835.8 | 12.3 | 28.0 | 38.3 | 31.3 | 40.0 | 24.4 |
|  | Mean | 854.7 | 10.6 | 29.0 | 34.5 | 26.1 | 41.1 | 23.2 |
|  | SD | 17.9 | 1.3 | 2.2 | 2.9 | 3.9 | 1.2 | 0.8 |
|  | CV | 2.1 | 12.1 | 7.6 | 8.3 | 14.8 | 3.0 | 3.6 |
|  |  |  |  |  |  |  |  |  |
| 8 | CV0067 | 675.9 | 14.8 | 23.0 | 18.9 | 14.5 | 20.7 | 25.6 |
| 8 | CV0067_Dup | 625.1 | 15.7 | 17.8 | 19.3 | 14.1 | 19.5 | 22.9 |
| 9 | CV0067_Rep | 536.8 | 11.6 | 22.1 | 19.6 | 10.5 | 15.7 | 20.5 |
|  | Mean | 612.6 | 14.0 | 21.0 | 19.3 | 13.0 | 18.6 | 23.0 |
|  | SD | 57.5 | 1.8 | 2.3 | 0.3 | 1.8 | 2.1 | 2.1 |
|  | CV | 9.4 | 12.6 | 10.9 | 1.4 | 14.0 | 11.4 | 9.2 |
|  |  |  |  |  |  |  |  |  |
| 9 | CV0091 | 428.7 | 7.0 | 21.3 | 26.8 | 9.6 | 29.8 | 14.6 |
| 9 | CV0091_Dup | 455.7 | 7.5 | 22.3 | 31.0 | 10.0 | 31.8 | 16.1 |
| 10 | CV0091_Rep | 490.9 | 7.7 | 22.5 | 27.4 | 8.2 | 36.6 | 16.4 |
|  | Mean | 458.4 | 7.4 | 22.0 | 28.4 | 9.2 | 32.7 | 15.7 |
|  | SD | 25.4 | 0.3 | 0.6 | 1.9 | 0.7 | 2.8 | 0.8 |
|  | CV | 5.6 | 4.1 | 2.6 | 6.6 | 8.1 | 8.7 | 5.0 |
|  |  |  |  |  |  |  |  |  |
| 10 | CV0949 | 262.4 | 4.8 | 17.4 | 24.8 | 5.4 | 41.0 | 20.5 |
| 10 | CV0949_Dup | 256.4 | 6.0 | 17.7 | 26.6 | 5.4 | 41.4 | 21.3 |
| 11 | CV0949_Rep | 318.3 | 7.5 | 24.0 | 24.5 | 5.4 | 47.0 | 24.0 |
|  | Mean | 279.0 | 6.1 | 19.7 | 25.3 | 5.4 | 43.1 | 21.9 |
|  | SD | 27.9 | 1.1 | 3.0 | 0.9 | 0.0 | 2.7 | 1.5 |
|  | CV | 10.0 | 18.2 | 15.5 | 3.5 | 0.3 | 6.3 | 6.8 |


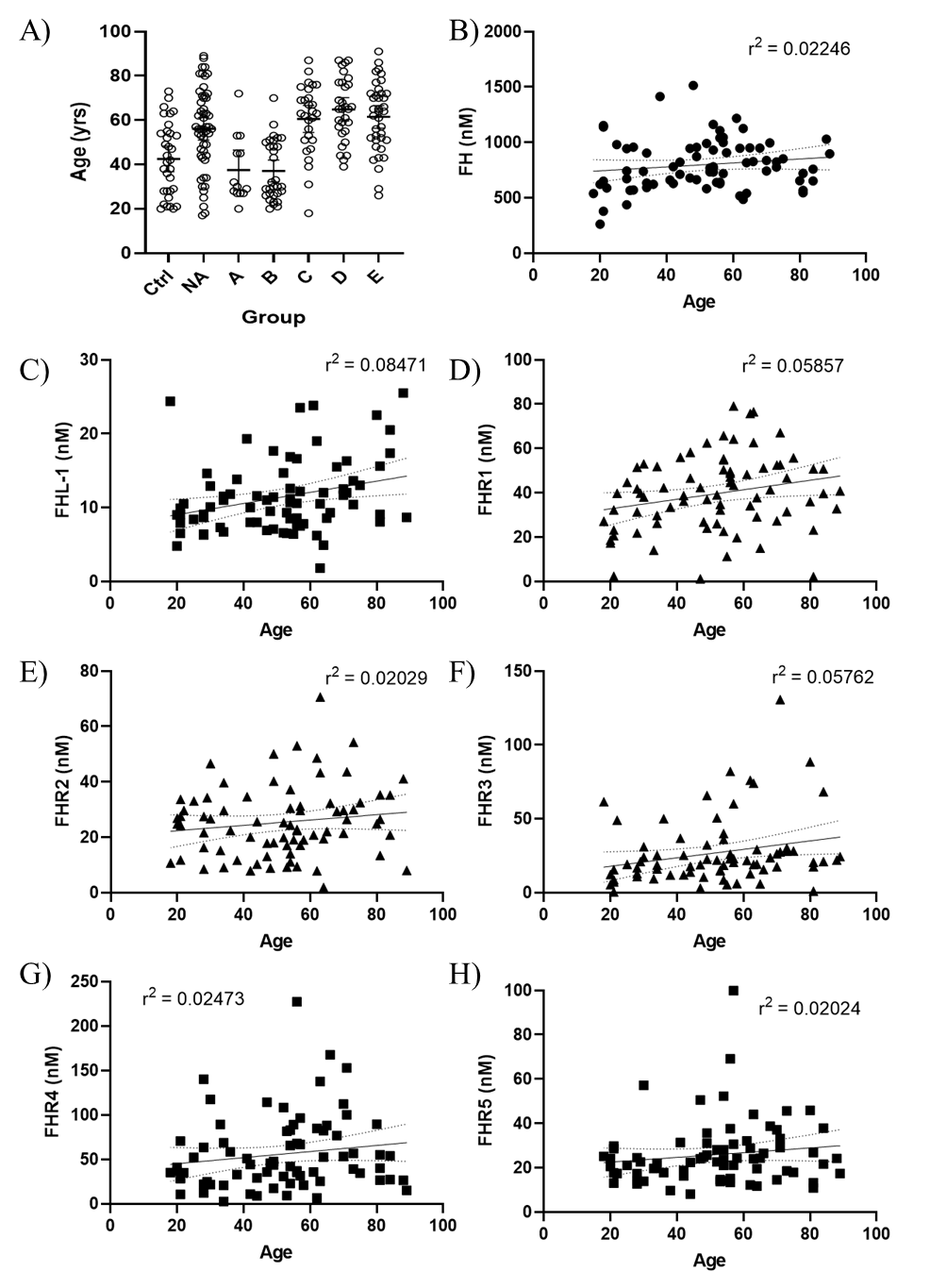


**Supplementary Figure 2.** **Correlation with age of FH, FHL-1 and FHR1-5 in all non-COVID-19 samples (total n=76).** A) Distribution of age within each experimental group. All between-group differences where p<0.01 (One-way ANOVA) are marked. B-H) Linear regression line is shown with 95% confidence intervals, with Spearman’s r^2^ value displayed on each graph

**
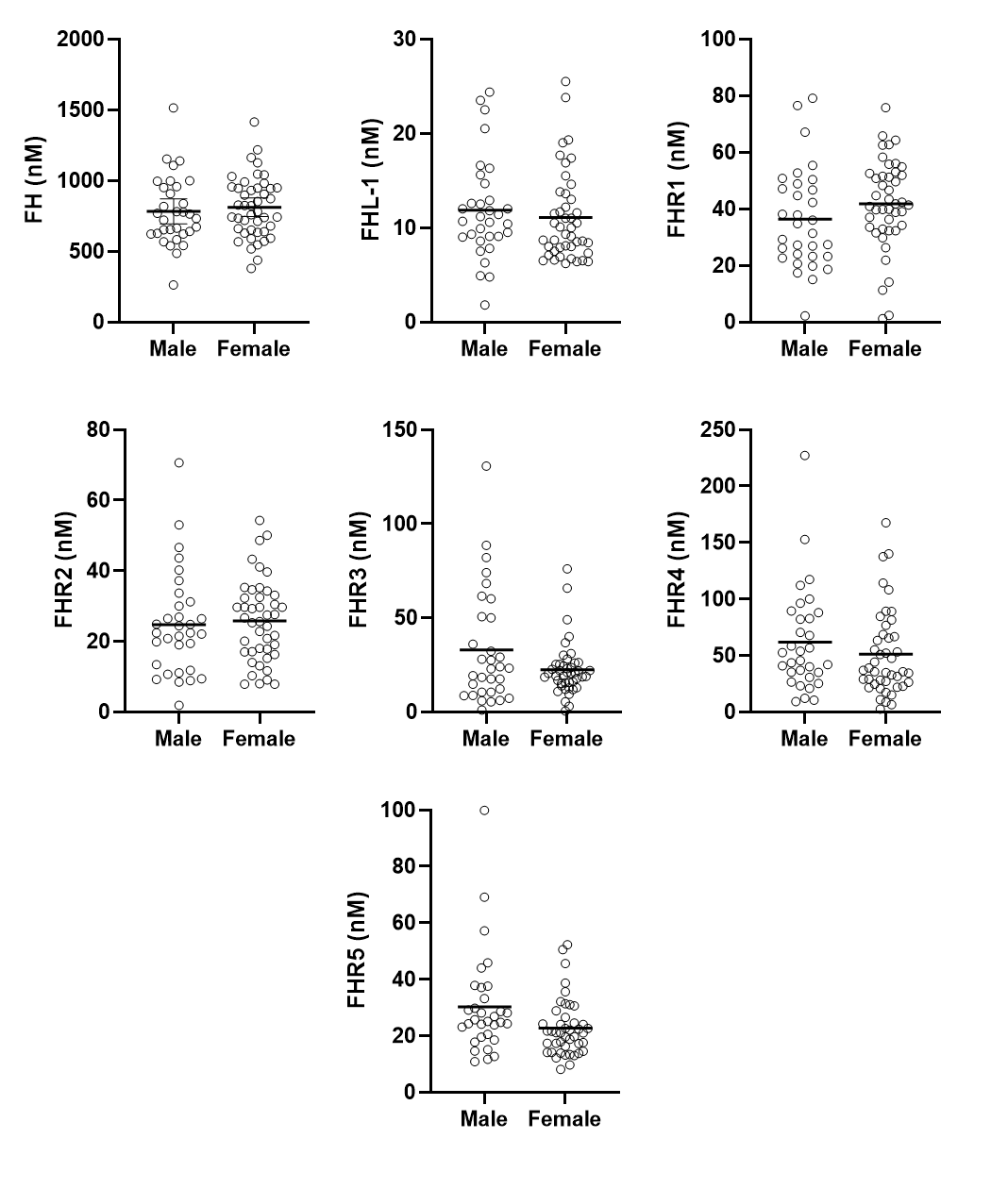
Supplementary Figure 3.** **Correlation of sex with circulating FH, FHL-1 and FHR1-5 in all non-COVID-19 samples (total n=76).** Data are shown as scatter plots with the mean marked. None of the compatrisons reached statistical significance by a Mann-Witney U test.


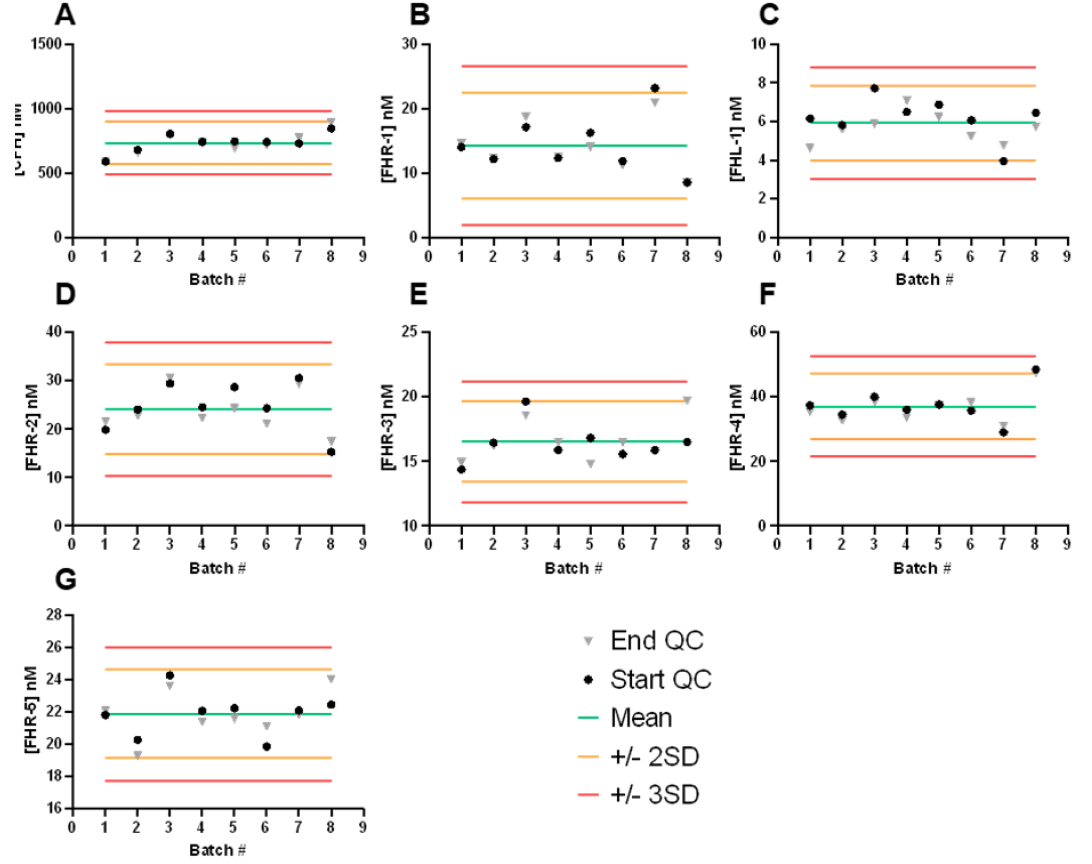


**Supplementary Figure 4.** **Levey-Jennings graphs illustrating the QC results to monitor the between-batch stability of the whole process across 8 batches for the analysis of 154 baseline and timecourse samples.** Duplicate QC samples were included in each batch, and the concentration of each peptide was measured.

**
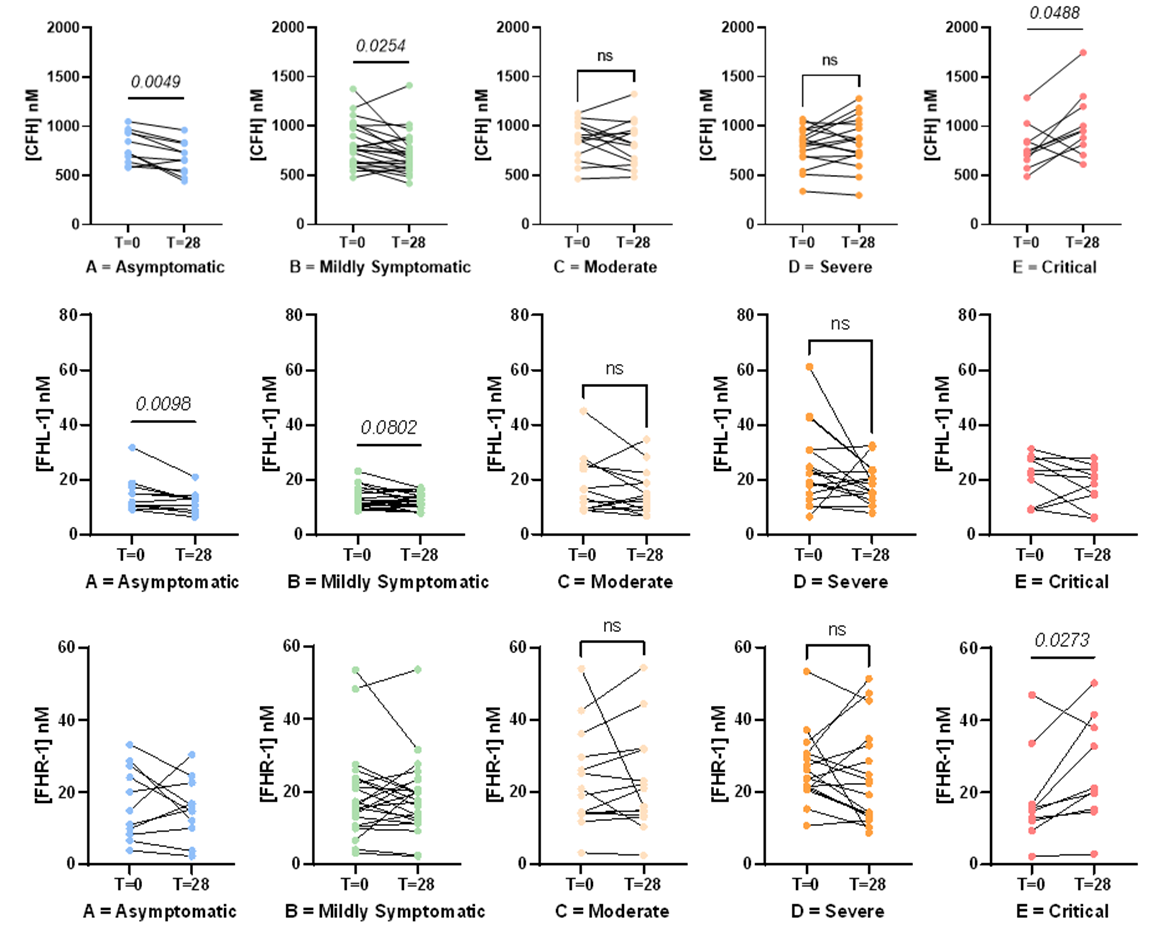
Supplementary Figure 5A-C. Trajectory of circulating levels** of A) CFH, B) FHL-1, and c) FHR1 in a baseline sample and a 28-day sample for individuals testing positive for COVID-19, separated by disease severity. P-value is calculated using a Wilcoxon-signed rank test and is provided to 4 d.p. Only comparisons where p<0.05 are shown.

A)

B)

C)

**
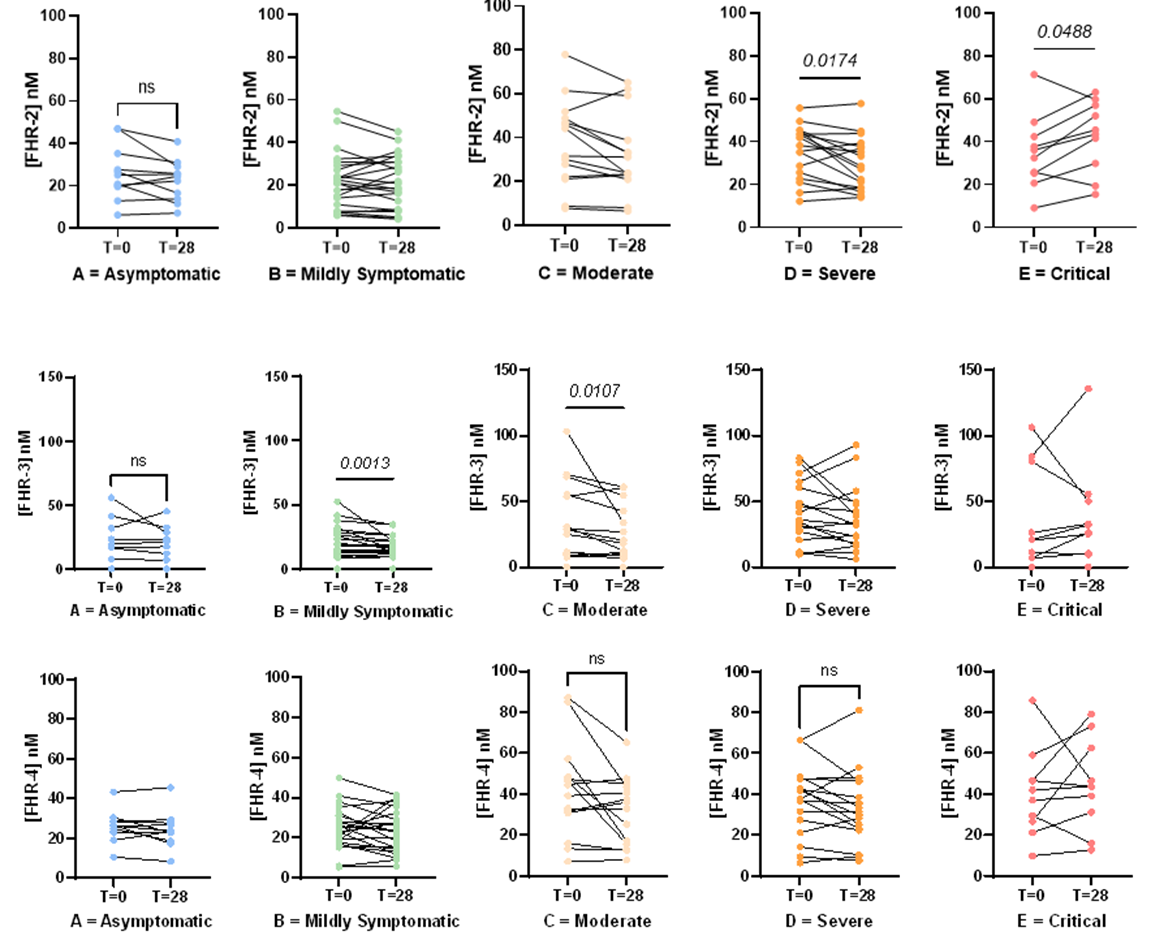
**

**Supplementary Figure 5D-F. Trajectory of circulating levels** of D) FHR2, E) FHR3, and F) FHR4 in a baseline sample and a 28-day sample for individuals testing positive for COVID-19, separated by disease severity. P-value is calculated using a Wilcoxon-signed rank test and is provided to 4 d.p. Only comparisons where p<0.05 are shown.

D)

E)

F)

**
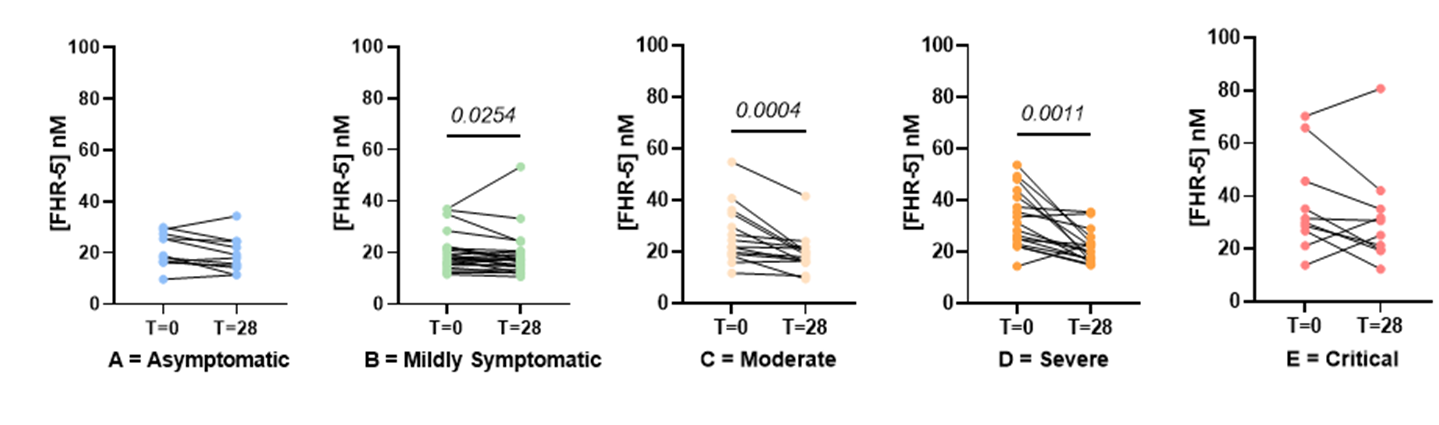
**

**Supplementary Figure 5G. Trajectory of circulating levels** of G) FHR5 in a baseline sample and a 28-day sample for individuals testing positive for COVID-19, separated by disease severity. P-value is calculated using a Wilcoxon-signed rank test and is provided to 4 d.p. Only comparisons where p<0.05 are shown.

G)

RD
