# Supplementary material for: Levels of soluble complement regulators predict severity of COVID-19 symptoms
Source: Front Immunol. 2022 Oct 18;13:1032331. doi: 10.3389/fimmu.2022.1032331 (PMC9624227; doi:10.3389/fimmu.2022.1032331)
Supplement: Supplementary file 2 [file DataSheet_2.docx]

**The CITIID-NIHR BioResource COVID-19 Collaboration Authorship Banner**

Stephen Baker^2,6^, John Bradley^1,3,6,11,15^ , Patrick Chinnery^3,23,24^, Daniel Cooper^11, 25^,

Gordon Dougan^2,6^, Ian Goodfellow^7^, Ravindra Gupta^2,6,13,16^, Nathalie Kingston^3,4^, Paul J. Lehner^2,6,13^, Paul A. Lyons^2,6^, Nicholas J. Matheson^2,6,13,33^, Caroline Saunders^9^, Kenneth G. C. Smith^2,6^, Charlotte Summers^6,12,26^, James Thaventhiran^19^, M. Estee Torok ^6,13,14^, Mark R. Toshner^6,8,26^, Michael P. Weekes^2,6,13,34^, Gisele Alvio^9^, Sharon Baker^9^, Areti Bermperi^9^, Karen Brookes^9^, Ashlea Bucke, Jo Calder, Laura Canna, Cherry Crucusio, Isabel Cruz^9^, Ranalie de Jesus^9^, Katie Dempsey^9^, Giovanni Di Stephano^9^, Jason Domingo^9^, Anne Elmer^9^, Julie Harris, Sarah Hewitt, Heather Jones^9^, Sherly Jose^9^, Jane Kennet, Yvonne King, , Jenny Kourampa^9^, Emily Li, Caroline McMahon^9^, Anne Meadows, Vivien Mendoza^9^, Criona O’Brien, Charmain Ocaya^9^, Ciro Pasquale^9^, Marlyn Perales^9^, Jane Price, Rebecca Rastall, Carla Ribeiro^9^, Jane Rowlands, Valentina Ruffolo, Hugo Tordesillas, Phoebe Vargas^9^, Bensi Vergese^9^, Laura Watson^9^, Jieniean Worsley^9^, Julie-Ann Zerrudo^9^, Laura Bergamashi^2,6^, Ariana Betancourt, Georgie Bower, Ben Bullman, Chiara Cossetti, Aloka De Sa, Benjamin J. Dunmore, Maddie Epping, Stuart Fawke, Stefan Gräf ^3,6^, Richard Grenfell, Andrew Hinch, Josh Hodgson, Christopher Huang, Oisin Huhn, Kelvin Hunter^2,6^, Isobel Jarvis, Emma Jones, Maša Josipović, Ekaterina Legchenko, Daniel Lewis, Joe Marsden, Jennifer Martin, Federica Mescia^2,6^, Ciara O’Donnell, Ommar Omarjee, Marianne Perera, Linda Pointon, Nicole Pond, Nathan Richoz, Nika Romashova, Natalia Savoinykh, Rahul Sharma, Joy Shih, Mateusz Strezlecki, Rachel Sutcliffe, Tobias Tilly, Zhen Tong, Carmen Treacy, Lori Turner, Jennifer Wood, Marta Wylot, John Allison^3,4^, Heather Biggs^3,18^, John R. Bradley^1,3,6,11,15^, Helen Butcher^3,5^, Daniela Caputo^3,5^, Matt Chandler^3,5^, Patrick Chinnery^3,23,24^, Debbie Clapham-Riley^3,5^, Eleanor Dewhurst^3,5^, Christian Fernandez^3,^ Anita Furlong^3,5^, Barbara Graves^3,5^, Jennifer Gray^3,5^, Sabine Hein^3,5^, Tasmin Ivers^3,5^, Emma Le Gresley^3,5^, Rachel Linger^3,5^, Mary Kasanicki^3,11^, Rebecca King^3,5^, Nathalie Kingston^3,4^, Sarah Meloy^3,5^, Alexei Moulton^3,5^, Francesca Muldoon^3,5^, Nigel Ovington^3,4^, Sofia Papadia^3,5^, Christopher J. Penkett^3,4^, Isabel Phelan^3,5^, Venkatesh Ranganath^3,4^, Roxana Paraschiv^3,4^, Abigail Sage^3,5^, Jennifer Sambrook^3,4^, Ingrid Scholtes^3,5^, Katherine Schon^3,17,18^, Hannah Stark^3,5^, Kathleen E. Stirrups^3,4^, Paul Townsend^3,4^, Neil Walker^3,4^, Jennifer Webster^3,5^, Mayurun Selvan^35^, Petra, Polgarova^12^,Sarah L. Caddy^2,6^, Laura G. Caller^20,21^, Yasmin Chaudhry^7^, Martin D. Curran^22^, Theresa Feltwell^6^, Stewart Fuller^20^, Iliana Georgana^7^, Grant Hall^7^, William L. Hamilton^6,13,14^, Myra Hosmillo^7^, Charlotte J. Houldcroft^6^, Rhys Izuagbe^7^, Aminu S. Jahun^7^, Fahad A. Khokhar^2,6^, Anna G. Kovalenko^7^, Luke W. Meredith^7^, Surendra Parmar^22^, Malte L. Pinckert^7^, Anna Yakovleva^7^, Emily C. Horner^19^, Lucy Booth^19^, Alexander Ferreira^19^, Rebecca Boston^19^, Robert Hughes^19^, Juan Carlos Yam Puc^19^, Nonantzin Beristain-Covarrubias^19^, Maria Rust^19^, Thevinya Gurugama^19^, Lihinya Gurugama^19^, Thomas Mulroney^19^, Sarah Spencer^19^, Zhaleh Hosseini^19^, Kate Williamson^19^.

^1^NIHR Cambridge Biomedical Research Centre, Cambridge Biomedical Campus, Cambridge, UK
^2^Cambridge Institute of Therapeutic Immunology and Infectious Disease (CITIID), Jeffrey Cheah Biomedical Centre, Cambridge Biomedical Campus, Cambridge, UK

^3^NIHR BioResource, Cambridge University Hospitals NHS Foundation Trust, Cambridge Biomedical Campus, Cambridge, UK

^4^Department of Haematology, School of Clinical Medicine, University of Cambridge, Cambridge Biomedical Campus, Cambridge, UK

^5^Department of Public Health and Primary Care, School of Clinical Medicine, University of Cambridge, Cambridge Biomedical Campus, Cambridge, UK

^6^Department of Medicine, School of Clinical Medicine, University of Cambridge, Cambridge Biomedical Campus, Cambridge, UK

^7^Division of Virology, Department of Pathology, University of Cambridge, Cambridge, UK

^8^Royal Papworth Hospital NHS Foundation Trust, Cambridge, UK

^9^Cambridge Clinical Research Centre, Addenbrooke’s Hospital, Cambridge University Hospitals NHS Foundation Trust, Cambridge, UK

^10^Intensive Care Unit, Royal Papworth Hospital NHS Foundation Trust, Cambridge, UK

^11^Addenbrooke’s Hospital, Cambridge University Hospitals NHS Foundation Trust, Cambridge Biomedical Campus, Cambridge, UK

^12^Intensive Care Unit, Addenbrooke’s Hospital, Cambridge University Hospitals NHS Foundation Trust, Cambridge Biomedical Campus, Cambridge, UK

^13^Department of Infectious Diseases, Addenbrooke’s Hospital, Cambridge University NHS Hospitals Foundation Trust, Cambridge, UK

^14^Department of Microbiology, Addenbrooke’s Hospital, Cambridge University NHS Hospitals Foundation Trust, Cambridge, UK

^15^Department of Renal Medicine, Addenbrooke’s Hospital, Cambridge University Hospitals NHS Foundation Trust, Cambridge, UK

^16^Africa Health Research Institute, Durban, South Africa

^17^Clinical Genetics, Addenbrooke’s Hospital, Cambridge University Hospitals NHS Foundation Trust, Cambridge, UK

^18^Department of Clinical Neurosciences, School of Clinical Medicine, University of Cambridge, Cambridge Biomedical Campus, Cambridge, UK

^19^MRC Toxicology Unit, Gleeson Building, Tennis Court Road, Cambridge, UK

^20^University of Cambridge, Cambridge, UK

^21^The Francis Crick Institute, London, UK

^22^Public Health England, Clinical Microbiology and Public Health Laboratory, Cambridge, UK

^23^Department of Clinical Neurosciences, School of Clinical Medicine, University of Cambridge, Cambridge Biomedical Campus, Cambridge, UK

^24^Medical Research Council Mitochondrial Biology Unit, Cambridge Biomedical Campus, Cambridge, UK

^25^Global and Tropical Health Division, Menzies School of Heath Research and Charles Darwin University, Darwin, Northern Territory, Australia

^26^Heart and Lung Research Institute, Cambridge Biomedical Campus, Cambridge, UK

^27^Department of Rheumatology, Addenbrooke’s Hospital, Cambridge University Hospitals NHS Foundation Trust, Cambridge, UK

^28^Cambridge Cancer Trials Centre, Addenbrooke’s Hospital, Cambridge University Hospitals NHS Foundation Trust, Cambridge, UK

^29^Department of Paediatrics, University of Cambridge, Cambridge Biomedical Campus, Cambridge, UK

^30^Patient Safety, Addenbrooke’s Hospital, Cambridge University Hospitals NHS Foundation Trust, Cambridge, UK

^31^Clinical Research Network: Eastern, Addenbrooke’s Hospital, Cambridge University Hospitals NHS Foundation Trust, Cambridge, UK

^32^Institute of Metabolic Science, Addenbrooke’s Hospital, Cambridge University Hospitals NHS Foundation Trust, Cambridge, UK

^33^NHS Blood and Transplant, Cambridge, UK

^34^Cambridge Institute for Medical Research, Biomedical Campus, Hills Rd, Cambridge UK

^35^ Department of Respiratory Medicine, Cambridge University Hospitals NHS Foundation Trust, Cambridge, UK
